# Long-term follow-up on the use of vascularized fibular graft for the treatment of congenital pseudarthrosis of the tibia

**DOI:** 10.1186/1749-799X-3-13

**Published:** 2008-03-06

**Authors:** Akio Sakamoto, Tatsuya Yoshida, Yoshio Uchida, Tetsuo Kojima, Hideaki Kubota, Yukihide Iwamoto

**Affiliations:** 1 Department of Orthopaedic Surgery, Graduate School of Medical Sciences, Kyushu University, Fukuoka, Japan; 2 Uchida Orthopaedic Surgery Hospital, Fukuoka, Japan; 3 Mizoguchi Orthopaedic Surgery Hospital, Fukuoka, Japan; 4 Saga Handicapped Children's Hospital, Saga, Japan

## Abstract

**Background:**

Congenital pseudoarthrosis of the tibia (CPT) is one of the most difficult conditions to treat.

**Methods:**

Five girls and 3 boys with CPT were treated by vascularized fibular grafting (VFG). The average age at VFG was 7.0 years (range: 1.9–11.5 years) with an average follow-up term of 11.7 years (range: 4.9–19.6 years). Five of the children had undergone multiple operations before VFG, while the other 3 had no such history.

**Results:**

Bone consolidation was obtained in all cases after an average term of 6.6 months (range: 4–10 months); this was with the first VFG in 7 cases but with the second VFG in 1 case. Complication of stress fracture and ankle pain occurred in 1 and 3 cases, respectively, only in cases undergoing multiple operations. Leg-length discrepancy was more prominent in the patients with multiple previous operations (mean: 7.5 cm), than in the cases with no prior surgery (mean: 0.7 cm).

**Conclusion:**

The long-term results of VFG for CPT were excellent, especially in the cases, with no prior surgery. VFG should be considered as a primary treatment option for CPT.

## Background

Congenital pseudoarthrosis of the tibia (CPT) is one of the most difficult conditions to treat. The natural history is persistent instability and progressive deformity [1,2]. CPT is known to accompany NF1 (neurofibromatosis type 1), also called von Recklinghausen disease. Treatment options vary, including both surgical and non-surgical approaches. Surgical techniques of vascularized fibular grafting (VFG), intramedullary stabilization and external fixation have been reported to be relatively successful in the treatment of CPT [3-9].

We previously reported the cases of 5 patients with CPT for whom good short-term results were obtained with the use of VFG [3]. However, long-term follow-up studies of VFG, particularly identifying limb-length discrepancy, residual angular deformity and the rates of refracture are necessary. All of those complications can compromise the functional outcome, even though pseudarthrosis may demonstrate bone consolidation [2,10]. In this study, the long-term results of VFG were evaluated for 5 previously reported cases and for an additional 3 cases. We specifically emphasize a comparison between patients undergoing multiple operations and those with no prior surgery before VFG. The previous surgerical procedures were in the current series were all intramedullary stabilization with/without bone grafting, which was not accompanied by any method of microvascular bone transplantation.

## Methods

This is a retrospective review of the clinical results in 8 patients with CPT managed with VFG performed by Y.U. or T.K. at Kyushu University Hospital (Table 1). The patients comprised 5 girls and 3 boys. Six of them had NF1 (6/8; 75%). The tibia with pseudarthrosis involved the right side in 5 cases and the left side in 3 cases. Ipsilateral VFG was applied as a first choice, and contralateral VFG was undertaken when ipsilateral fibula was not available. Consequently, ipsilateral VFG was applied in 7 cases and contralateral VFG was applied in 2 cases, in which one was for the initial trial of VFG, and the other was for the second trial of VFG after failure of bone consolidation in ipsilateral VFG.

**Table 1:** Vascularized bone-transferred cases with/without previous multiple operations

Case/Side/NF1	Sex/Age	Number of previous operations	Donor site	Term until union	Age at follow-up (term)	Leg-length discrepancy (before VFG)	Residual angulations anterior/valgus	Stress fracture (after VFG)	Corrective osteotomy	Ankle pain (after VFG)
Cases with previous multiple operations										
1/L/+	F/7.4 yo	8	I	9 m	27.0 yo (19.6 y)	5.2 cm (5.0)	28/10 deg	+ (4 m)	-	+ (12 y)
2/L/+	F/8.8 yo	3	I	6 m	21.6 yo (12.8 y)	5.8 cm (6.0)	21/0 deg	-	-	+ (9 y)
3/L/+	F/8.8 yo	7	I	5 m	27.3 yo (19.0 y)	10.2 cm (9.0)	5/3 deg	-	-	-
4/R/-	M/8.9 yo	4	C	5 m	22.2 yo (13.3 y)	0.6 cm (0.0)	18/2** deg	-	+	+ (11 y)
5/R/+	F/11.5 yo	6	I	9 m	18.5 yo (7.0 y)	15.7 cm (14.2)	0/0 deg	-	-	-
Cases without prior surgery										
6/R/-	M/1.9 yo	0	I	Non union						
	/7.3 yo	1*	C	5 m	14.8 yo (7.4 y)	0.0 cm	0/0** (22/20) deg	-	+	-
7/R/+	M/3.2 yo	0	I	10 m	8.1 yo (4.9 y)	0.0 cm	20/20 deg	-	-	-
8/R/+	F/6.2 yo	0	I	4 m	15.3 yo (9.1 y)	2.0 cm	0/0 deg	-	-	-

*; First trial of vascularized bone-transferred operations, **; data after corrective osteotomy, NF1; neurofibromatosis type 1, VFG; vascularized fibular grafting, L; left, R; right, M; male, F; female, yo; years old, y; years, m; months, I; ipsilateral, C; contralateral, ant; anterior, valg; valgus, deg; degrees.

Multiple previous operations had been performed before VFG in 5 patients in other institutions prior to attending our hospital. We have reported these patients in a study of short-term follow-up [3]. For these patients, the number of operations ranged from 3 to 8, the average number being 3.4. The previous surgical treatments were all utilizing intramedullary nails with/without bone grafting. The other 3 patients were added for the purpose of the current study, and they had undergone no previous treatments. We analyzed the term of bone consolidation, and complications comprising leg-length discrepancy, tibial deformity of angulation, occurrence of fractures and existence of ankle pain, in a comparison between patients with and without previous surgical treatment before VFG. Bone consolidation was analyzed by skilled orthopedic surgeons including some of the authors.

### Operative technique

The method of VFG is summarized as follows: Before operation, the vascular anatomy was determined by angiography. Dissection of the vascularised (peroneal vessels) fibula was performed. The fibula proximal to the pseudoarthrosis site was usually used for the donor. The thick fibrous tissue around the tibial pseudarthrosis was resected completely, whereas resection of the sclerotic bone ends was minimal. After correction of angular deformity, a slot was created to receive the fibular graft which was secured by several screws. End-to-end anastomosis was performed between the anterior tibial and the peroneal vessels [3].

### Statistical analysis

Clinical data were statistically analyzed using the Mann-Whitney *U*-test for quantitative data of the term of bone consolidation and leg-length discrepancy, and using Fisher's exact test for qualitative data of the existence of ankle pain. A p value of less than 0.05 was considered to indicate statistical significance.

## Results

### Patients

The mean age of the patients at the time of VFG surgery was 7.0 years old (ranging from 1.9 to 11.5 years old). Average postoperative follow-up term was 11.7 years (ranging from 4.9 to 19.6 years), and the average patient age at final follow-up was 19.3 years old (ranging from 8.1 years to 27.3 years old). Recurrence was not seen in any of the cases during the course of the follow-up.

### Bone consolidation

**Figure 1:**
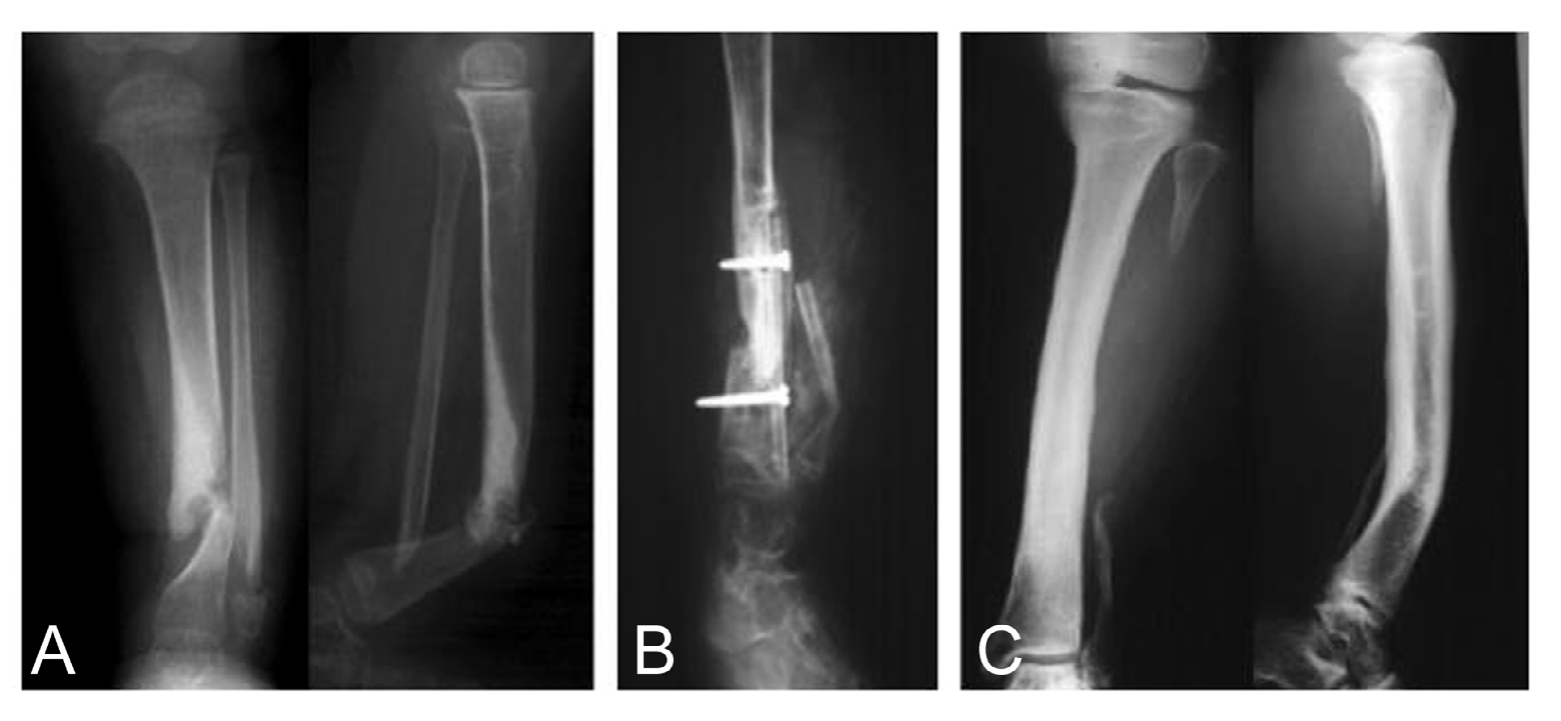
Congenital pseudarthrosis of the tibia (Case 1; a case undergoing multiple operations). Eight operations was undergone before VFG at 2.5 years old (A), Ipsilateral VFG was performed at 7.4 years old (B). At 23.5 years old, 15 years after VFG (C).

**Figure 2:**
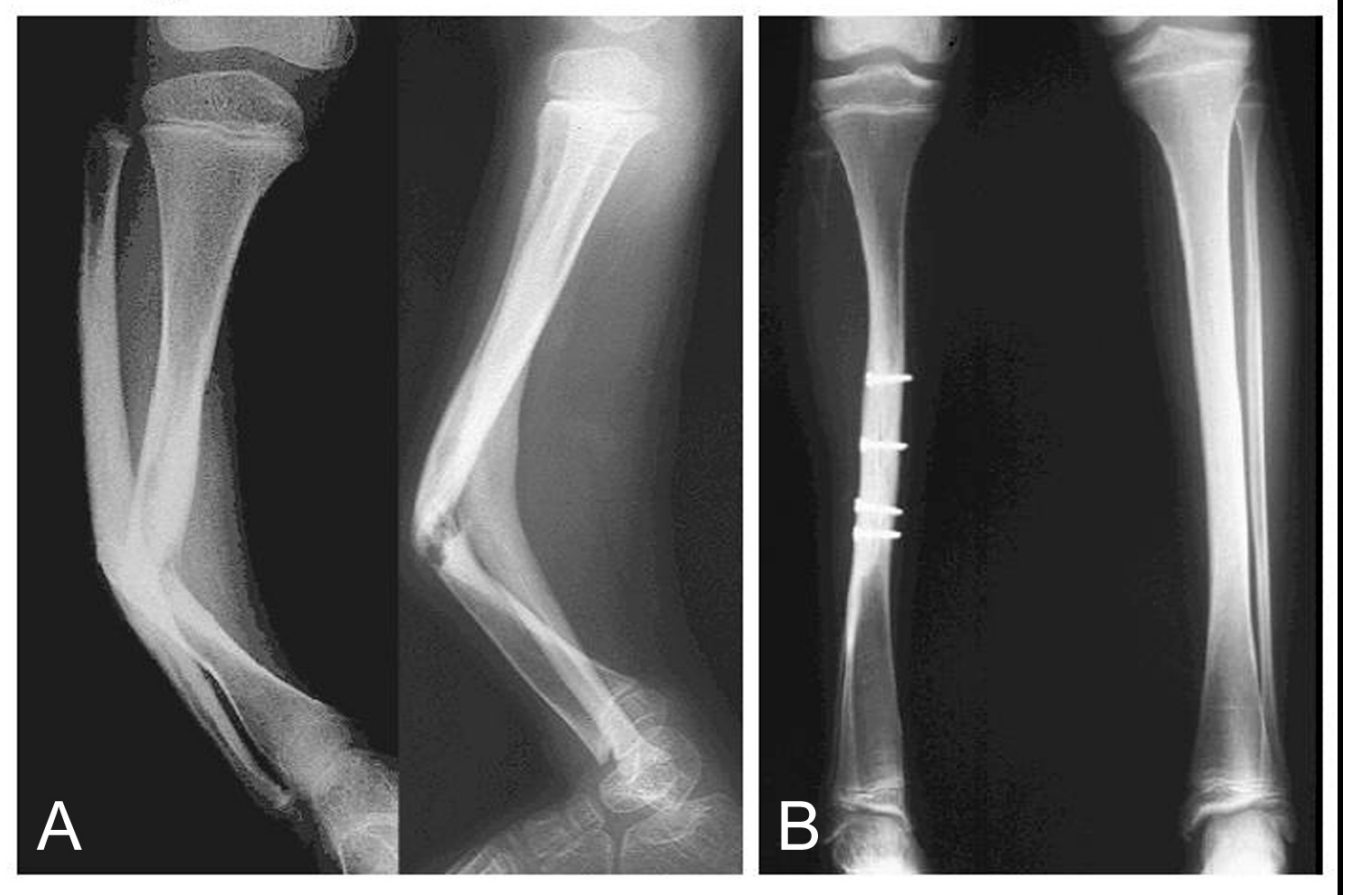
Congenital pseudarthrosis of the tibia (Case 8; a case with no prior surgery) with no other operation before VFG. Preoperation status at 6.6 years old (A). At 12.8 years old, 6.2 years after VFG (B).

**Figure 3:**
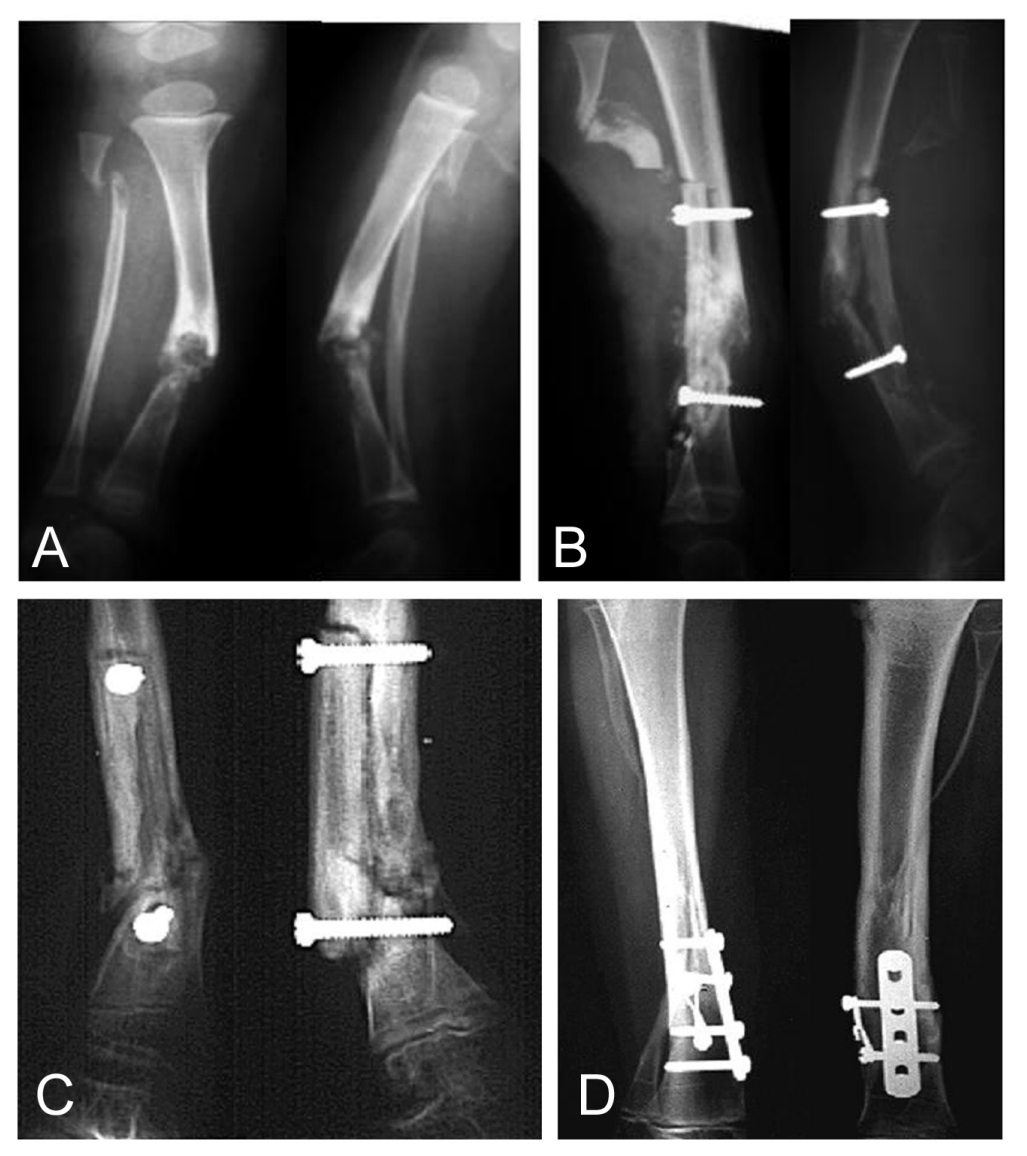
Congenital pseudarthrosis of the tibia (Case 6; a case with no prior surgery). Preoperation status of VFG (A). Ipsilateral VFG was performed at 1.9 years old (B). At 7.3 years old, bone consolidation can be seen 5 months after second VFG from the contralateral side (C). At 14.8 years old, after corrective osteotomy of the lower tibia for the deformity (D).

Bone consolidation after VFG occurred in all the patients (8/8: 100%). In the patients undergoing multiple operations, all 5 cases obtained bone consolidation after the first VFG operation without any further surgery (Figs. 1, 2), while 1 out of the 3 cases without prior surgery failed to obtain bone consolidation following the first ipsilateral VFG at the age of 1.9 years old. A second contralateral VFG was then undertaken for this patient at the age of 7.3 years old, and bone consolidation was obtained 5 months after the second operation (Case 6; Fig. 3). Counting the second VFG in this case as data, the bone consolidation term of all 8 cases averaged 6.6 months (ranging from 4 to 10 months), in which bone consolidation was obtained 7 months after the second operation in this case. In the cases with no prior surgery, two out of the 3 patients obtained bone consolidation within 10 months. The bone consolidation term for the patients undergoing multiple operations was 6.8 months, while that for patients with no prior surgery was 6.3 months. There was no significant difference between these results (p = 0.77).

### Complications

#### Leg-length discrepancy

The overall average postoperative discrepancy in all cases was 4.9 cm (ranging from 0 to 15.7 cm). The leg-length discrepancy for the cases undergoing multiple operations was 7.5 cm (ranging from 0.6 to 15.7 cm), while that of cases with no prior surgery was 0.7 cm (ranging from 0.0 to 2.0 cm) (p = 0.07). A discrepancy of more than 5.0 cm was seen in 4 out of the 5 cases undergoing multiple operations, but in none of the 3 cases with no prior surgery. For cases undergoing multiple operations, leg-length discrepancy with an average of 6.8 cm (ranging from 0.0 to 14.2 cm) already existed. Therefore there was no significant difference between the previous state (6.8 cm) and the post state (7.5 cm) of VFG in the group of cases undergoing multiple operations (p = 0.84). The discrepancy seemed to be related to the earlier surgical procedures prior to VFG.

Tibial deformity of angulationDeformity of angulation varied, with the tibial valgus ranging from almost zero to 28.0 degrees and anterior bowing ranging from almost zero to 20.0 degrees. Deformity of more than 20 degrees occurred in 4 out of the 8 patients (4/8; 50%), of which 2 were cases undergoing multiple operations (2/5; 40%) and 2 were cases with no prior surgery (2/3; 67%). Two cases had undergone corrective osteotomy, of which one had undergone multiple operations and the other had undergone no prior surgery (Cases 4, 6).

#### Fractures

One patient had a stress fracture 4 months after VFG (Case 1). The fracture was treated with a brace and it healed.

#### Ankle pain

Ankle pain was seen in 3 out of the 8 cases, regardless of its severity. These pains appeared 12 years (at 19 years old), 9 years (at 17 years old) and 11 years (at 19 years old) after the surgery. The average term was 10.6 years and the average age was 18.3 years old. It seemed to be characteristic that these pains appeared at late adolescence. These cases of ankle pain seemed to be associated with the degree of tibial angulation. Moreover, ankle pain was seen in 3 out of the 5 cases undergoing multiple operations, but in none of the 3 cases with no prior surgery (p = 0.08).

#### Other factors

Gender or the existence of NF1 did not seem to have any relationship with bone consolidation or any other complications.

## Discussion

In a series of VFG for the treatment of CPT, bone consolidation was reported to be obtained in 94% of cases [4]. In the current study, all cases with VFG obtained bone consolidation, with an average bone consolidation term of 6.6 months without recurrence. In a previous report, gender may have been a significant factor in the length of term needed for bone consolidation, on the basis that 13 boys had an average bone consolidation term of 13 months, whereas 16 girls had an average bone consolidation term of 9 months [2]. In the current case, such a tendency was not observed. In our institute, because of good results of bone consolidation after VFG, VFG has been chosen as the primary treatment, with the Ilizarov bone transport method being an alternative choice. In a previous report, the Ilizarov bone transport method was reported as being useful in achieving primary healing in CPT, but complications of refracture and postoperative deformities may occur [11]. Further examination of long-term follow-up after the Ilizarov bone transport method is necessary.

As for VFG, it has been reported that age is an important factor in the result of VFG, with regard to bone consolidation. In a previous report, seven patients operated on at 10 years of age or older had successful outcomes, compared with 12 out of 22 who were 9 years of age or younger at the time of surgery [12]. According to the EPOS (European Paediatric Orthopaedic Society) Multicenter Study [6], there was a clear correlation between age at surgery and final outcome, with better results being achieved with increasing age. Therefore, it has been proposed that surgery should not be performed on patients younger than the age of 3 years and it is recommended that surgery be postponed until the age of 5 years [6]. Another study about bone consolidation in CPT also suggested that the best age for rapid bone consolidation is 3.5 years to 7.5 years old [4]. In the current study, one case aged 1.9 years old did not obtain bone consolidation with VFG. For that case, a second contralateral VFG was successful at the age of 7.3 years old. This fact may support the notion that an age younger than 3 or 3.5 years old is a negative factor with regard to bone consolidation in VFG.

Tibial deformities of limb-length discrepancy and angulation are common after treatment for CPT [2,4]. Bone consolidation of pseudarthrosis is not sufficient for assessment as the end result. Occasionally, chronic lower-extremity dysfunction and clinical symptoms may result in amputation [7]. In the current series, a limb-length discrepancy of more than 5 cm was seen in 4 out of the 5 cases undergoing multiple operations but in none of the 3 cases with no prior surgery. The average leg-length discrepancy for the cases undergoing multiple operations was 7.5 cm, while that of cases with no prior surgery was 0.7 cm. The p value is 0.07, and the reason for there being no significant difference statistically may be because of the small number of these cases. Limb-length discrepancy has been reported in half the patients with intramedullary nails [7]. Such surgical procedures with a potential danger of damaging the growth plate may result in limb-length discrepancy. Therefore, the deformity may have been related to an earlier surgical procedure prior to VFG [12].

Angular deformities do not remodel and are often progressive after VFG [5,13]. In the current study, a deformity of more than 20 degrees was seen in 4 out of the 8 cases. The relationship between the degree of angulation and multiple operations before VFG is not clear. Ankle pain was seen in 3 out of 8 cases. It seemed characteristic that these pains appeared long after the VFG (mean, 10.6 years), and late in the second decade (mean, 18.3 years old). These 3 cases had undergone multiple operations. Therefore, it should perhaps be noted that there was a tendency for previous unoperated cases to have ankle pain at long-term follow-up, even though there had been no pain of short-term follow-up. In a previous report, ankle pain after the surgical procedure for VFG is associated with multiple operations utilizing intramedullary nails, consistent with our results [14], and degenerative changes in the ankle because of the ankle valgus deformity and the intramedullary rod passing through the ankle joint is considered to be the cause of the ankle pain [14-16]. Refracture is not uncommon following consolidation of VFG [13,17,18]. In most cases, the first fracture is reported to occur before the age of 1 year [4]. In the current case, one out of the 8 cases had a stress fracture at the age of 7.4 years old, and casting healed the fracture.

## Conclusion

In conclusion, the overall long-term follow-up results of VFG were excellent. However, residual limb-length discrepancy and ankle pain were prominent in cases undergoing multiple operations. In contrast, patients who underwent VFG as a primary operation had fewer such problems. Accordingly, VFG should be considered as a primary treatment option for CPT.

## Abbreviations

CPT; congenital pseudarthrosis of the tibia, NF1; neurofibromatosis type 1, VFG; vascularized fibular graft, LLD; leg-length discrepancy

## Competing interests

The author(s) declare that they have no competing interests.

## Authors' contributions

**AS **drafted the manuscript. **YU **and **TK **performed vascularized fibular graft. **AS**, **TY **and **HK **participated in the design of the study. **YI **conceived of the study, and participated in its design and coordination and helped to draft the manuscript. All authors read and approved the final manuscript.
